# Afatinib Reverses EMT via Inhibiting CD44-Stat3 Axis to Promote Radiosensitivity in Nasopharyngeal Carcinoma

**DOI:** 10.3390/ph16010037

**Published:** 2022-12-27

**Authors:** Huichao Huang, Fangling Huang, Xujun Liang, Ying Fu, Zhe Cheng, Yan Huang, Zhuchu Chen, Yankun Duan, Yongheng Chen

**Affiliations:** 1 Department of Infectious Disease, XiangYa Hospital, Central South University, Changsha 410008, China; 2 Department of Oncology, NHC Key Laboratory of Cancer Proteomics & State Local Joint Engineering Laboratory for Anticancer Drugs, National Clinical Research Center for Geriatric Disorders, Xiangya Hospital, Central South University, Changsha 410008, China; 3 Department of Hyperbaric Oxygen, Xiangya Hospital, Central South University, Changsha 410008, China

**Keywords:** afatinib, radiosensitivity, epithelial-to-mesenchymal transition (EMT), CD44-Stat3 signaling pathway, nasopharyngeal carcinoma (NPC)

## Abstract

Background: Afatinib, a second-generation tyrosine kinase inhibitor (TKI), exerts its radiosensitive effects in nasopharyngeal carcinoma (NPC). However, the detailed mechanism of afatinib-mediated sensitivity to radiation is still obscure in NPC. Methods: Quantitative phosphorylated proteomics and bioinformatics analysis were performed to illustrate the global phosphoprotein changes. The activity of the CD44-Stat3 axis and Epithelial-Mesenchymal Transition (EMT)-linked markers were evaluated by Western blotting. Wound healing and transwell assays were used to determine the levels of cell migration upon afatinib combined IR treatment. Cell proliferation was tested by CCK-8 assay. A pharmacological agonist by IL-6 was applied to activate Stat3. The xenograft mouse model was treated with afatinib, radiation or a combination of afatinib and radiation to detect the radiosensitivity of afatinib in vivo. Results: In the present study, we discovered that afatinib triggered global protein phosphorylation alterations in NPC cells. Further, bioinformatics analysis indicated that afatinib inhibited the CD44-Stat3 signaling and subsequent EMT process. Moreover, functional assays demonstrated that afatinib combined radiation treatment remarkably impeded cell viability, migration, EMT process and CD44-Stat3 activity in vitro and in vivo. In addition, pharmacological stimulation of Stat3 rescued radiosensitivity and biological functions induced by afatinib in NPC cells. This suggested that afatinib reversed the EMT process by blocking the activity of the CD44-Stat3 axis. Conclusion: Collectively, this work identifies the molecular mechanism of afatinib as a radiation sensitizer, thus providing a potentially useful combination treatment and drug target for NPC radiosensitization. Our findings describe a new function of afatinib in radiosensitivity and cancer treatment.

## 1. Introduction

Nasopharyngeal carcinoma (NPC), a malignancy derived from the nasopharyngeal epithelium, has a much higher incidence in Southern China, Southeast Asia and Northern Africa [1,2]. Epstein–Barr virus (EBV) infection, genetic susceptibility, and environmental factors are the main etiologic factors contributing to the tumorigenesis and development of NPC. Due to the anatomic constraints for surgery, the primary treatments for NPC include radiation therapy, chemotherapy, targeted drug therapy and immunotherapy [3,4]. Although great progress has been made in treatment, such as high-precision RT techniques, there is still a huge percentage of patients developing resistance to radiation, which cause local recurrence and distant metastases [5]. Therefore, it is urgent to explore effective sensitizers to IR for NPC patients.

Currently, the main approaches to improve the efficacy of radiotherapy are using chemotherapeutic and targeted drugs, such as 5-fluorouracil and platinum analogs [6,7]. However, several pre-clinical and clinical trials reported that these chemotherapeutic drugs did not achieve better outcomes than radiotherapy alone [8,9]. Targeted drugs, due to their prominent efficacy, precise treatment and minor side effects, have earned promising interest to be potential radiosensitizers [10]. In our previous study, afatinib, a new TKI for both EGFR and HER2, was demonstrated that augmented the radiation response in NPC cells [11]. However, how does afatinib improve radiosensitivity, which signaling pathway or biological process is involved? These are the fundamental questions that needed to be investigated further.

Epithelial-to-mesenchymal transition (EMT) is a reversible process with the characteristic of transition of epithelial cells to mesenchymal state. It is featured by the loss of epithelial markers, including E-cadherin, and the gain of mesenchymal markers such as N-cadherin, vimentin and fibronectin, which leads to functional changes in cell migration, invasion and therapeutic resistance, including radioresistance [12,13]. An array of signaling pathways is reported to regulate the EMT-mediated radioresistance in several types of tumors, containing TGF-β, Wnt/β-catenin, PI3K/AKT, IL-6/STAT3, EGFR and ERK [14,15,16,17]. Besides EMT, cancer stem cells (CSCs) property is another crucial factor that contributed to tumor cells’ resistance to radiation treatment. More importantly, EMT and CSCs usually crosstalk with each other to induce radioresistance. For example, research demonstrated that the EMT process strengthened the capability of CSCs phonotype in NSCLC [18]. Monica et al. reported that ALDH promoted DNA repair capacity and EMT process via the Wnt/β-catenin signaling pathway and contributed to tumor radioresistance in prostate cancer [15]. Meanwhile, a study in nasopharyngeal carcinoma showed that silencing FOXO3a led to acquired radioresistance by inducing EMT and activating the Wnt signaling pathway [19].

CD44 is a transmembrane glycoprotein and regulates cancer development and progression, which is overexpressed in many types of tumors and closely associated with a worse prognosis [20]. In addition, CD44, as a marker of CSCs and a vital regulatory factor in the EMT program, has a key role in mediating cancer metastasis and therapeutic resistance through activating and modulating several cell signaling networks [21]. For example, in breast cancer cells, ESRP1 regulated the expression of CD44v and silencing ESRP1 in CD44v+ cells led to a switch from CD44v to CD44s isoform, leading to inhibition of lung cancer metastasis [22]. By contrast, another study indicated that a shift in CD44 expression from CD44v to the CD44s was necessary for EMT and required for breast cancer progression [23]. Similar to the EMT process, CD44 is also attributed to radiation resistance of cancer cells. For instance, osteopontin (OPN) elevated the stem cell-like properties and radiation resistance by activating CD44 in adjacent glioma cells [24]. Similarly, in pancreatic cancer CD44s isoform was significantly increased upon high-dose X-ray irradiation and eventually caused resistance to radiation treatment via ERK phosphorylation and EMT process [25]. At the same time, CD44 interacted with *P300* and *SIRT1* led to *MDR1* and *BCL-xl* gene expression and chemoresistance through the β-catenin signaling pathway in breast cancer cells [26]. Meanwhile, several studies have demonstrated that CD44 linked with Stat3 contributed to cancer initiation and development.

Signal transducer and activator of transcription 3 (Stat3), which is an important transcription factor, has been proved to take a vital effect in modulating cell proliferation, migration, invasion, and therapeutic resistance in several kinds of malignant tumors, such as head and neck cancer, ovarian cancer, and breast cancer [27,28]. Aberrant expression and activation of Stat3 intensively participated in the development and progression of tumors. The activity of Stat3 is tightly controlled by post-translational modifications (PTMs), in particular by serine and tyrosine phosphorylation. It was shown that protein tyrosine phosphatase receptor type D (*PTPRD*) reduced the resistance to radiation treatment via increasing the level of dephosphorylation of Stat3 and the radiation-induced autophagy in NPC cells [29]. Most likely, the study reported that RIPK4 suppressed the invasion and metastasis of HCC by inhibiting the phosphorylation of Stat3 and the EMT process [30].

Based on the current researches and our previous study, in the present work, we hypothesize that afatinib-mediated improvement of radiosensitivity induces globally reprogrammed signaling events by regulating the expression of signaling molecules including kinases. Consequently, the changes of protein phosphorylation level contribute to the radiation sensitization effect of afatinib in NPC cells. Using quantitative phosphorylated proteomics and bioinformatic analysis, we demonstrate that afatinib with IR treatment alters protein phosphorylation of crucial signaling in NPC cells. More importantly, we reveal that afatinib improves radiosensitivity via inhibiting the activity of CD44-Stat3 axis and reversing EMT process. Thus, our results may provide a novel effective therapy for NPC patients and present a useful resource for future studies investigating in-depth molecular mechanisms of afatinib acted as a radiation sensitizer.

## 2. Results

### 2.1. Profile of Phosphoproteomics Data upon Afatinib and IR Treatment in NPC Cells

**Figure 1:**
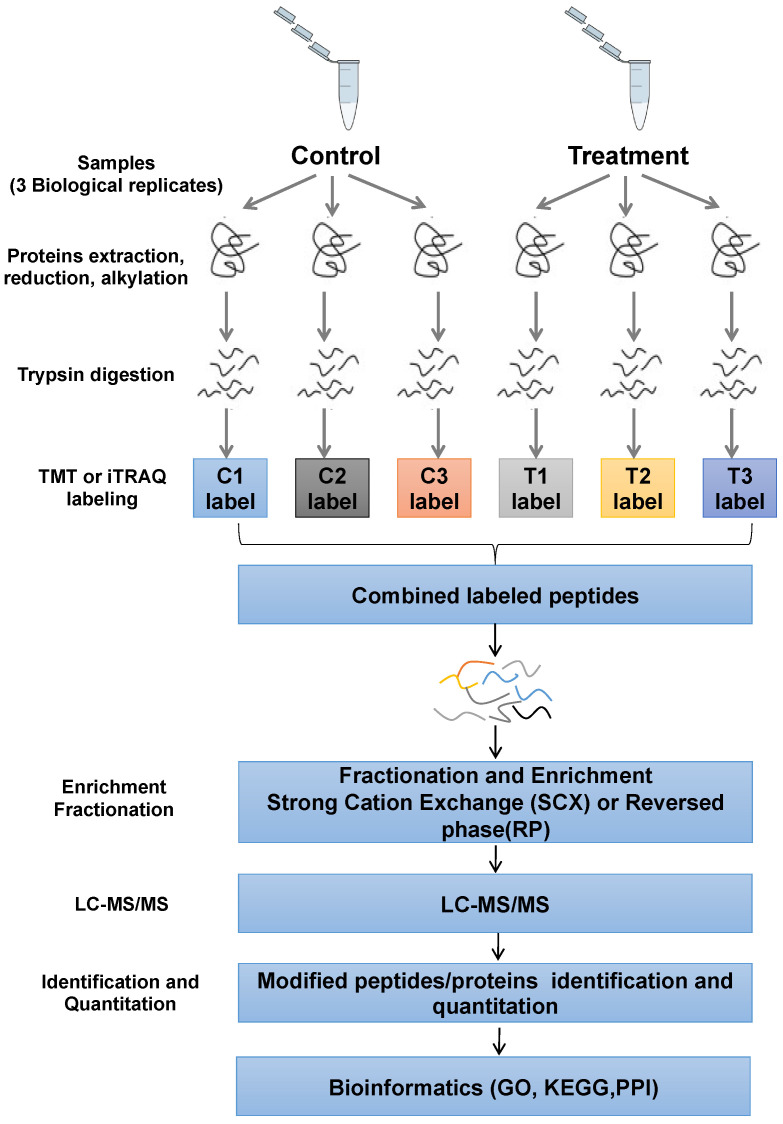
Schematic illustration of the TMT-based quantitative phosphoproteomic workflow. HNE2 cells pre-treated with IR or IR combined afatinib were conducted to TMT labeling, TiO_2_ enrichment and LC-MS/MS.

We have reported that afatinib, which simultaneously inhibited EGFR and HER2, augmented the radiosensitivity of NPC cells [11]. However, the underlying mechanism is still elusive, to explore the potential molecular mechanisms, we performed the quantitative phosphorylated proteomics of NPC cells pretreated with afatinib and IR or IR alone by 6-plex TMT. The workflow was shown in Figure 1.

**Figure 2:**
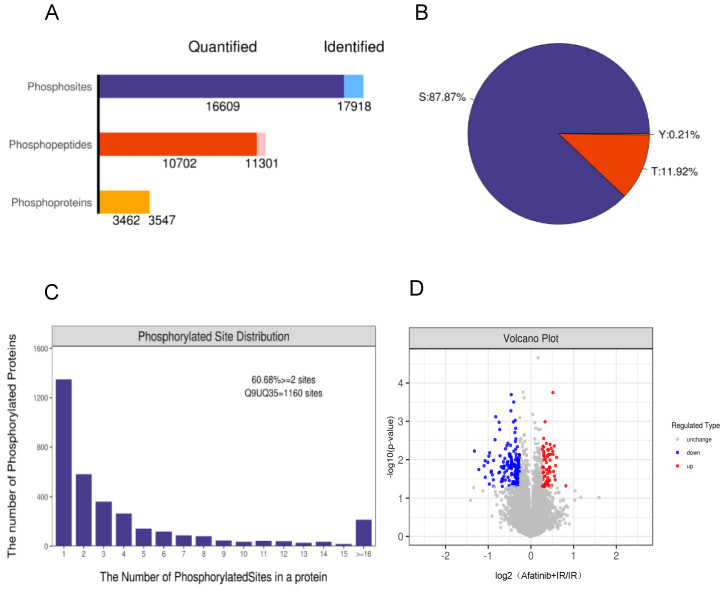
The distribution of phosphorylation sites. (**A**) Distribution of identified and quantified phosphorylated sites, phosphopeptides and phosphoproteins. (**B**) Distribution of phosphorylated sites on serine, threonine, and tyrosine. (**C**) Distribution of phosphorylated sites in a protein. (**D**) Volcano plots of group comparisons (Afatinib + IR vs. IR) showing the adjusted significance *p*-value (log2) versus fold change (log2). Red dots indicate significantly up-regulated phosphoproteins and blue dots indicate down-regulated.

Overall, 17,918 phosphorylated sites in 3547 phosphorylated proteins were identified, of which 16,609 phosphorylated sites in 3462 phosphorylated proteins were quantified (Figure 2A). 15,745 (87.87%) of the phosphorylation sites were found at serine, 2136 (11.92%) and 37(0.21%) located at threonine and tyrosine residues, respectively (Figure 2B). Among the identified 3547 phosphoproteins, 1395 (39.32%) phosphoproteins contained single phosphorylation, 2152 (60.68%) phosphoproteins had two or more than two phosphorylated sites (Figure 2C). Meanwhile, there were 170 phosphorylated proteins showed a significant change upon afatinib stimulation (*p* < 0.05; fold change > 1.2 or <−0.833), of which 52 phosphorylated proteins were up-regulated and 123 were down-regulated (Figure 2D). The top 20 differentially expressed phosphoproteins (DEPs) were displayed (Table 1).

### 2.2. Functional Characteristics and KEGG Pathway Analysis of DEPs

**Figure 3:**
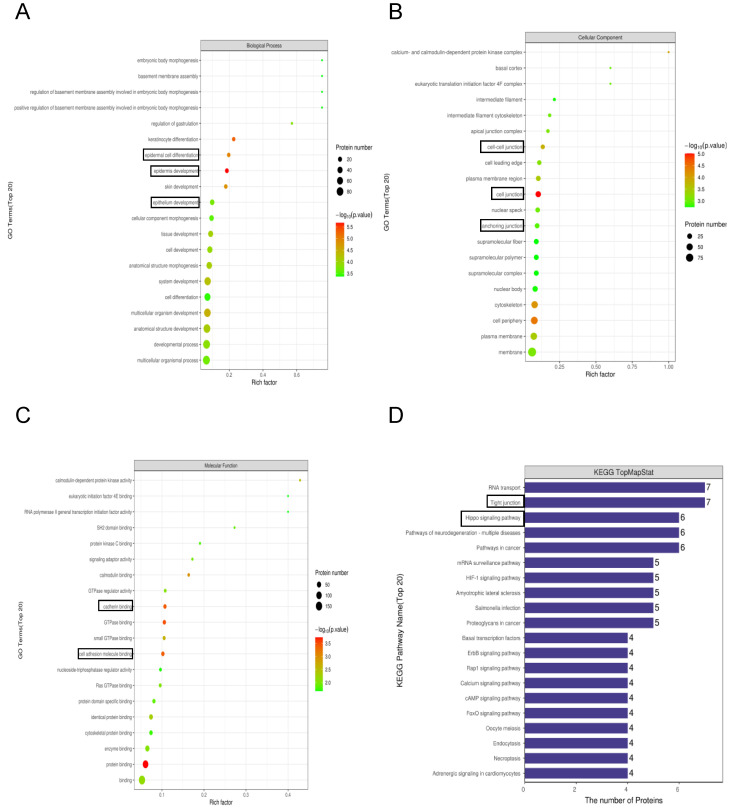
Functional characteristics and KEGG analysis of DEPs. (**A**) The biological processes analysis of DEPs. (**B**) The cellular components analysis of DEPs. (**C**) The molecular functions analysis of DEPs. (**D**) KEGG pathway enrichment analysis of DEPs.

To gain a whole insight into the cellular functions of the DEPs upon afatinib combined IR treatment, we conducted bioinformatics analysis to reveal the characteristic of protein domain based on GO, KEGG and interproscan database. First, the Gene Ontology (GO) based classification and enrichment analysis were analyzed. The biological process (BP) analysis indicated that the DEPs are closely related to the terms of epidermal cell differentiation, epidermis development and epithelium development (Figure 3A). The cellular component (CC) category implied that DEPs significantly participated in the cell–cell junction, cell junction and anchoring junction (Figure 3B). In the molecular function (MF) category, DEPs were markedly linked with cadherin binding and cell adhesion molecule binding (Figure 3C). Further, the KEGG pathway analysis revealed that cellular signaling pathways associated with EMT were significantly enriched such as tight junction, proteoglycans in cancer and the Hippo signaling pathway (Figure 3D). Collectively, these results indicated that afatinib may regulate the EMT process to augment the radiosensitivity of NPC cells.

### 2.3. CD44-STAT3 Axis Contributing to Affect the Radiosensitivity of NPC Cells

**Figure 4:**
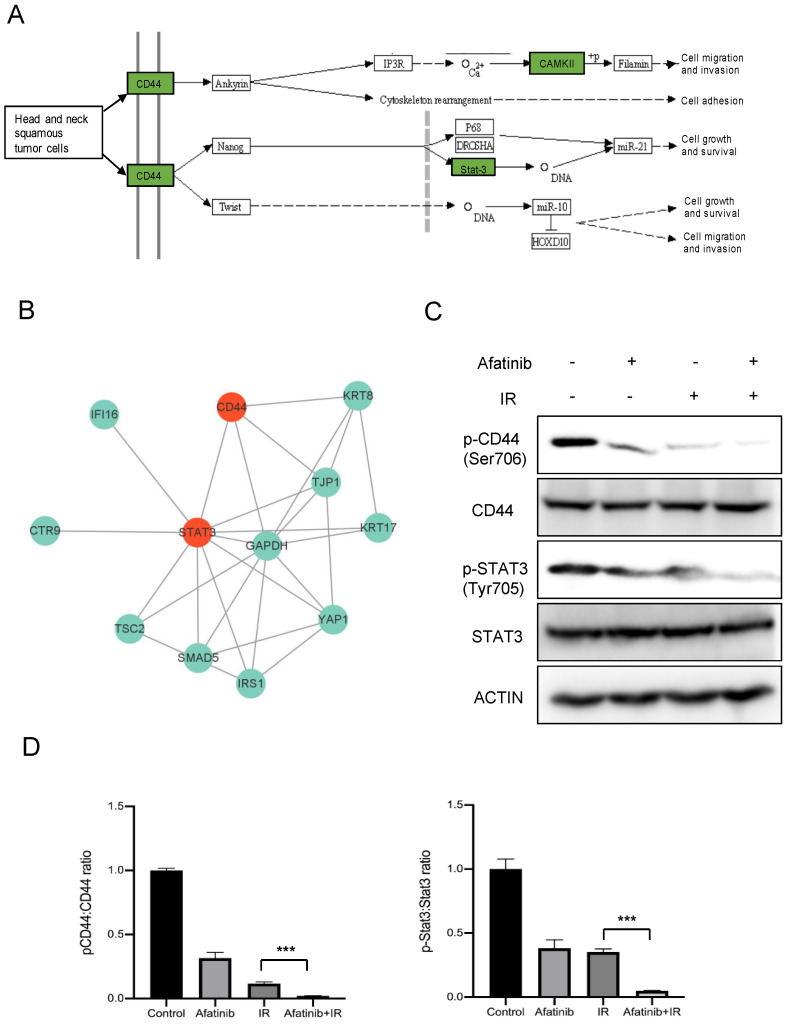
Phosphorylated proteomics analysis revealed influence on CD44-Stat3 axis. (**A**) Functions of the proteoglycans in cancer signaling pathway were presented. (**B**) DEPs interacted with CD44 and Stat3 were analyzed. (**C**,**D**) HNE2 cells were treated with afatinib or IR, WCLs were prepared at 24 h post-treatment and analyzed by immunoblotting with indicated antibodies (**C**). the statistical analysis is shown in (**D**). *** denotes *p* < 0.001. Error bars represent ±SD of triplicate experiments.

To determine the potential pathways to regulate the EMT process and radiosensitivity upon afatinib, we analyzed the DEPs and enriched signaling pathways, the result showed that “the proteoglycans in cancer signaling pathway” was highly related to the EMT process in Head and neck squamous tumor cells. In our study, five DEPs (CD44, Stat3, CAMK2D, CAMK2B and CAMK2G) were identified in the proteoglycans in cancer signaling pathway, which worked together to modulate functions of this pathway, such as cell migration and invasion, cell adhesion, cell growth and survival (Figure 4A). Moreover, CD44 and Stat3 acted as the top DEPs, and the expression and activity of which are correlated with EMT in different cancers. Thus, we asked whether the CD44-Stat3 axis was able to regulate radiosensitivity toward afatinib treatment in NPC cells.

To answer this question, we further explored interactive proteins among the identified DEPs with the candidate axis via STING and InAct database. As shown in Figure 4B, there were 10 DEPs interacted with CD44-Stat3 directly upon afatinib treatment, of which most proteins were taken part in the cell motility, cell migration and invasion processes. Furthermore, we validated activity of the CD44-Stat3 axis in response to IR and afatinib treatment in NPC cells. The results indicated that the phosphorylated signals of Ser706 in CD44 and Tyr705 in Stat3 were significantly reduced toward the combination of afatinib with IR treatment in HNE2 cells (Figure 4C,D), which further potentiated our hypothesis that the CD44-Stat3 axis affected radiosensitivity upon afatinib treatment in NPC cells.

### 2.4. Exploring the Inhibition of EMT Process in Combination with Afatinib and IR Treatment in NPC Cells

**Figure 5:**
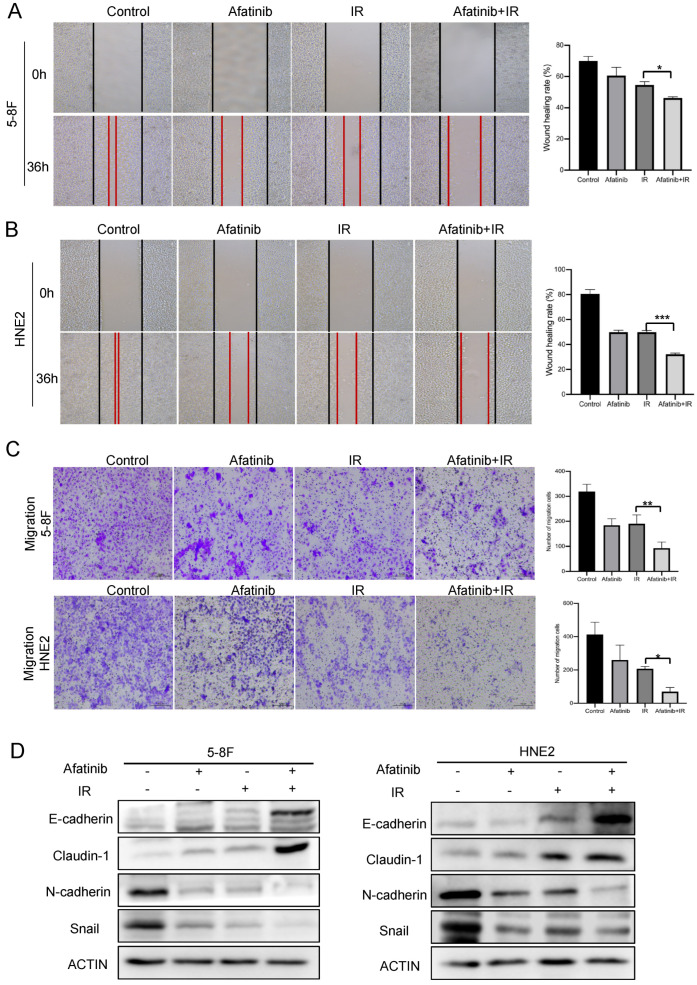
Afatinib combined IR treatment inhibited the EMT process. (**A**,**B**) 5-8F (**A**) and HNE2 cells (**B**) were treated as indicated and cell migration was measured by wound healing assay. (**C**) 5-8F and HNE2 cells were treated as indicated and cell migration was assessed by transwell assay without Matrigel. Cellular migration was plotted as the average number of cells in three different areas. (**D**) 5-8F and HNE2 cells were treated as indicated, WCLs were prepared at 24 h post-treatment and analyzed by immunoblotting with representative EMT marker antibodies. * denotes *p* < 0.05, ** denotes *p* < 0.01, *** denotes *p* < 0.001. Error bars represent ±SD of triplicate experiments.

EMT, as a crucial biological process, took part in cancer initiation, progression, metastasis, and drug resistance. Since the quantitative phosphorylated proteomics data indicated that afatinib with IR treatment manipulated the EMT process to augment radiation sensitivity in NPC cells. Next, we determined the capability of cell motility and classic EMT markers by wound healing, transwell and Western blotting assays. As observed in 5-8F and HNE2 cells, afatinib plus IR robustly suppressed the migration rate compared with IR treatment alone (Figure 5A,B). Similarly, transwell migration assay also displayed a markedly decreased cell migration in response to the afatinib combined IR treatment (Figure 5C). Meanwhile, the representative EMT-linked protein markers were validated via Western blotting assay. The data displayed that E-cadherin and Claudin-1, belonging to epithelial markers, were significantly increased, while N-cadherin and Snail, belonging to mesenchymal markers, were decreased upon afatinib and IR treatment (Figure 5D). Taken together, these findings demonstrated that afatinib treatment reversed the EMT process to augment radiosensitivity in NPC cells.

### 2.5. Stat3 Agonist Reversed Sensitivity to IR by Regulating EMT

The above results indicated that afatinib inhibited activity of the CD44-Stat3 axis via phosphorylation and regulated the EMT process to implement its enhanced radiosensitivity role. Further, in terms of our phosphorylated proteomics data and the previous studies, we considered that the CD44-Stat3 axis was responsible for the reversal of EMT upon afatinib and IR treatment in NPC cells.

**Figure 6:**
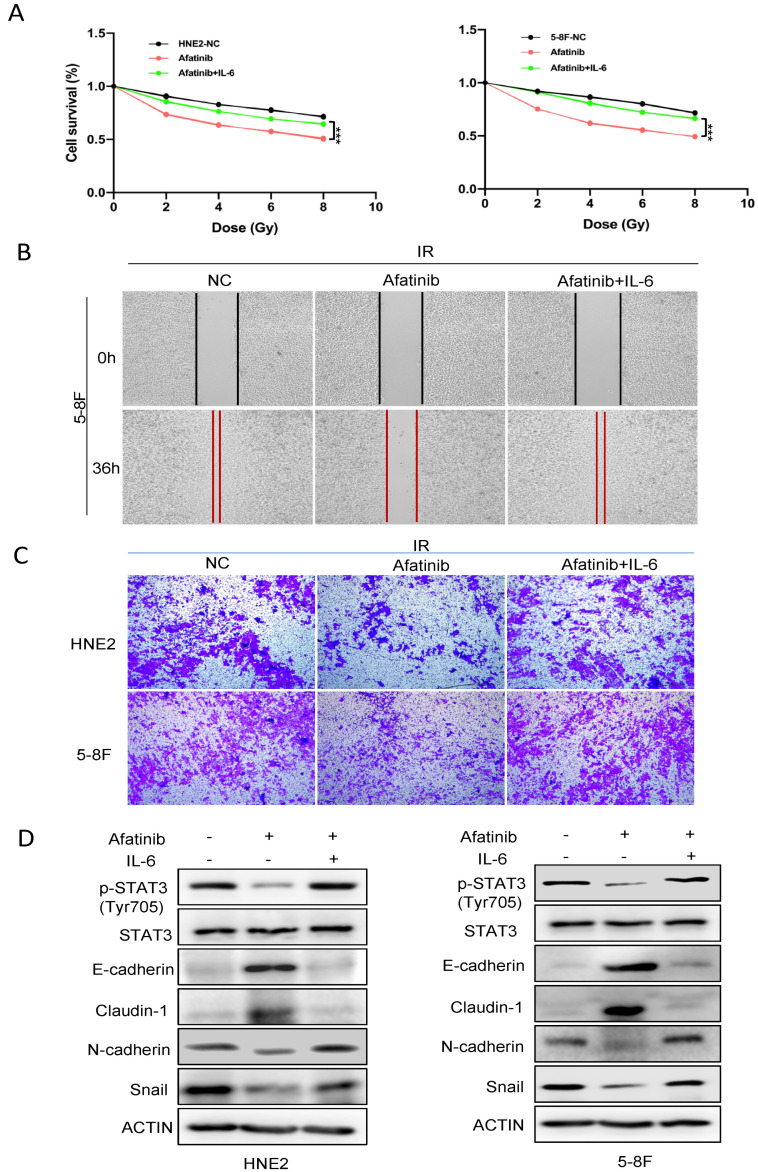
Stat3 agonist reversed sensitivity to IR by regulating EMT. (**A**) HNE2 cells were treated with or without IL-6 after exposure to afatinib combined IR treatment and cell viability was evaluated by CCK-8 assay. (**B**,**C**) HNE2 and 5-8F cells were treated with or without IL-6, cell migration was measured by wound healing assay (**B**) and transwell assay (**C**). (**D**) HNE2 and 5-8F cells were treated with IL-6 after exposure to IR, WCLs were prepared and analyzed by immunoblotting with indicated antibodies. *** denotes *p* < 0.001.

To further explore this question, the agonist of Stat3, IL-6, was introduced in NPC cells. Cell survival, migration possibility as well as EMT markers were assessed upon reactivation of Stat3 via IL-6. As presented in Figure 6A, the CCK-8 assay showed that p-Stat3 reactivation significantly attenuated cell survival in HNE2 and 5-8F cells compared to NC group toward afatinib and IR treatment. This result suggested that Stat3 activation could weaken the anti-tumor activity induced by afatinib. Furthermore, wound healing and transwell assays were conducted to examine cell migration. In agreement with our hypothesis, activation of Stat3 indeed improved cell migration ability both in HNE2 and 5-8F cells (Figure 6B,C). We then queried whether Stat3 reactivation could rescue the effect of afatinib. Both epithelial and mesenchymal markers were determined by Western blotting. We found that p-Stat3 reactivation abrogated increased expression of E-cadherin and Claudin-1, while improved that of N-cadherin and Snail caused by afatinib (Figure 6D), suggesting that reactivation of Stat3 promoted the EMT process. These results collectively supported that afatinib-mediated regulation of the CD44-Stat3-EMT axis was one of the prominent mechanisms underlying the enhanced radiation sensitivity in NPC cells.

### 2.6. Afatinib Enhanced Sensitivity to IR In Vivo

**Figure 7:**
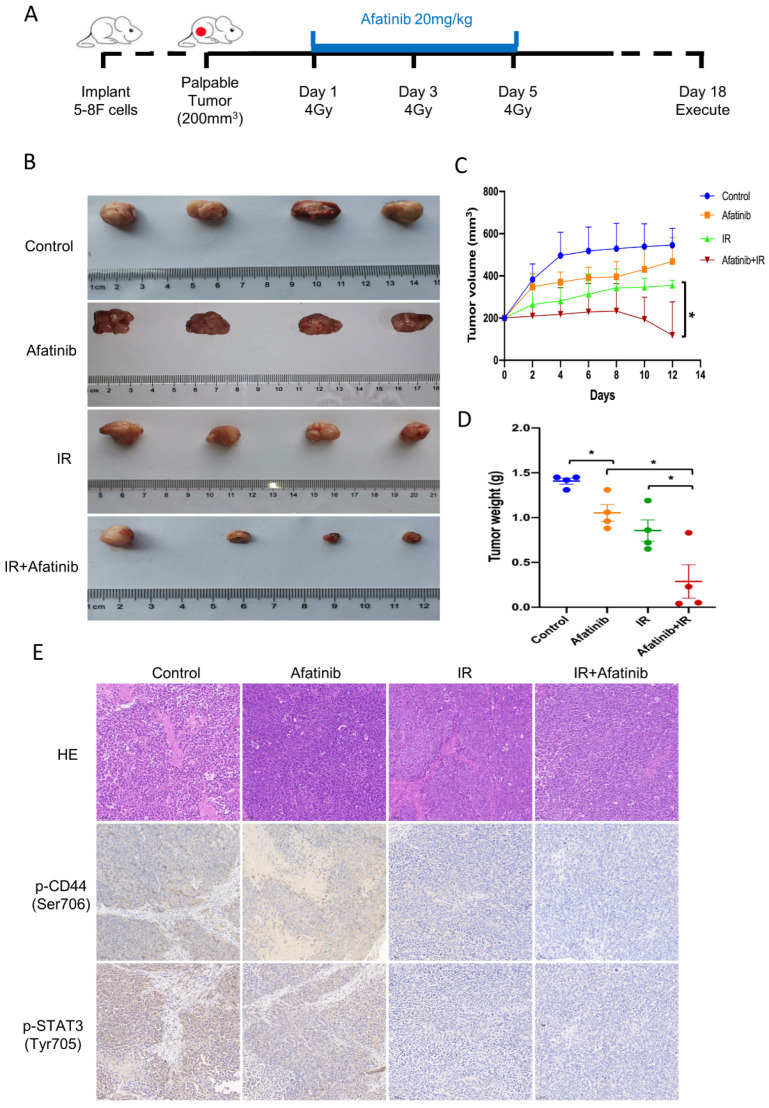
Afatinib enhanced sensitivity to IR in vivo. (**A**) Schematic of the xenograft mouse experiment. 5-8F cells were injected subcutaneously into the right flank of male nude mice. When tumor volume reached about 200 mm^3^, mice were induced with indicated treatments. After two weeks, the mice were executed, HE and IHC experiments were performed. (**B**) Representative images of dissected xenografts. (**C**,**D**) Tumor volume (**C**) and tumor weight curve (**D**). (**E**) Representative images of HE and IHC staining from xenografts. * denotes *p* < 0.05.

We next monitored whether afatinib augmented sensitivity to IR in vivo. The Xenograft mouse model was used, and the experiment scheme was displayed in Figure 7A. Consistent with our in vitro results, afatinib and IR treatment markedly suppressed tumor growth compared with IR inducement (Figure 7B). Tumor volume and tumor weight were analyzed, and the data demonstrated that both tumor volumes and weight in the afatinib combined IR group robustly decreased compared to that in the IR group (Figure 7C,D). The following phosphorylated signals of CD44 and Stat3 were assessed by IHC. We found that afatinib and IR treatment significantly impaired phosphorylated signals of CD44 and Stat3 (Figure 7E). These results confirmed that afatinib enhanced radiosensitivity in NPC xenograft models, which suggested that afatinib is a potential radiosensitizer for NPC patients.

## 3. Discussion

Ionizing radiation (IR) is still the criterion therapy for NPC patients, thus almost all the patients will receive IR treatment alone or in combination with chemotherapy during their disease course. However, resistance to IR is an urgent obstacle that brings about failure of treatment, and short of radiosensitizer is a crucial clinical challenge [4,5]. We have demonstrated previously that afatinib, an inhibitor of both EGFR and HER2, sensitized the radiation efficacy of NPC cells. Here, the underlying mechanism of the radiosensitization of afatinib is further explored, and we report that afatinib promotes radiosensitivity by reversing the EMT process via manipulating activity of the CD44-Stat3 axis in NPC cells. Moreover, utilizing the agonist of the CD44-Stat3 axis aggravates the transition of epithelial to mesenchymal, and improves cell survival and migration.

In the present study, we perform quantitative phosphorylated proteomics and identify a robust phosphorylated protein turnover in the IR combined afatinib treatment group. It suggests that globally reprogrammed signaling events via modulating the expression of signaling molecular containing kinase may have an important role in the enhancement of radiosensitivity of afatinib in the NPC cells. Based on the phosphorylated proteomics data, the phosphorylation of CD44 (Ser 706) and Stat3 (Tyr 705) are downregulated in the IR plus afatinib group compared to the IR treatment group, and the dysregulation of the CD44-Stat3 axis is significantly associated with the EMT process. Remarkably, augmented the activity of Stat3 by IL-6 inducement could inhibit the sensitivity to radiation caused by afatinib. Overall, our observation provides a novel insight that afatinib improves the therapeutic efficacy of radiotherapy via manipulating the activity of the CD44-Stat3 axis and EMT process in NPC cells, besides its intrinsic effects in inhibiting EGFR and HER2.

Various reports demonstrated that dysregulation of some cellular signalings via phosphorylation leads to oncogeneses, such as JAK/Stat3, PI3-kinase/Akt/mTOR, receptor tyrosine kinases/Ras/Raf/MEK/ERK and MEKK/MKK/JNK [31,32,33]. At the same time, phosphorylation and dephosphorylation play a crucial role in the regulation of the activities of CD44 and Stat3, and aberrant phosphorylation of CD44-Stat3 is linked with various tumor progressions, such as NPC, breast cancer and lung cancer [29,34]. Thus, exploring phosphorylated proteomes in cancer is vital for providing information on tumor signaling, as well as building the foundation for novel therapies.

According to the phosphoproteomics data, we identify the CD44-Stat3 axis, the phosphorylation of which is impeded toward afatinib and IR treatment, might play a pivotal role in afatinib enhancing radiosensitivity in NPC cells. Such a hypothesis is substantiated by the following observations. First, the bioinformatics analysis shows that the activity of CD44-Stat3 axis is robustly attenuated in the afatinib plus IR group, compared to IR treatment alone. Additionally, the functional analysis demonstrates that the CD44-Stat3 axis is closely associated with the EMT process, which participates in the resistance to radiation in various tumors, such as NPC, lung cancer and breast cancer. Second, we demonstrate that afatinib combined IR treatment impedes the phosphorylation of CD44 (Ser 706) and Stat3 (Tyr 705) both in 5-8F and HNE2 cells (Figure 4C). Furthermore, IL-6, the agonist of Stat3, could rescue the biological functions of afatinib, including cell viability, cell migration as well as the Epithelial-Mesenchymal transition, supporting the crucial role of Stat3 in regulating the radiation sensitivity of NPC cells upon afatinib inducement. Third, in vivo experiment shows that afatinib-associated IR treatment strongly suppresses the xenograft tumor growth compared with IR treatment (Figure 7B). Additionally, both p-CD44 (Ser 706) and p-Stat3 (Tyr 705) are further decreased in the afatinib combined IR group, which agrees with the results of in vitro experiments. Accumulating evidence indicates that the dysregulation of the CD44-Stat3 axis is involved in tumor progression, invasion, metastasis and therapeutic resistance. For breast cancer cells, IL-6/JAK/Stat3 signaling pathway is essential to maintain the CSCs property (CD44^+^CD24^−^ cells), which suggested a prospective therapy mean to target the stem-like breast cancer cells [35]. In urinary bladder cancer cell lines, downregulation of CD44v3 increases cell apoptosis and cell cycle via inhibiting p-STAT3 signal, while using an anti-CD44 antibody to block CD44 signaling could attenuate STAT3 activation in cultured atrial fibroblasts, hinting that blocking CD44-dependent signaling might be a therapeutic option for cancer treatment [36]. Another group demonstrated that blocking or silencing Stat3 increased the sensitivity to anoikis in NPC cells, besides that knockdown Stat3 also reserved tumor invasive properties and inhibited expression of CD44 [37]. Here, the current study shows that the CD44-Stat3 signaling pathway is robustly associated with tumor progression and radiation resistance. However, how afatinib regulates the CD44-Stat3 axis to augment the radiosensitivity of NPC cells still is an open question. Further investigation will certainly reveal the importance of afatinib in cellular and molecular biology, and importantly may provide more efficacy therapeutic targets for NPC patients.

EMT process is known for its viral functions in tumorigenesis, metastasis and therapeutic resistance. The findings that vitamin D conferred radiosensitivity by reversing EMT in CRC models [38], and EMT was correlated to the refractory response to immunotherapy in breast cancer [39], which supported the hypothesis that EMT may contribute to sensitizing therapeutic regimens of cancer cells. Further supporting this hypothesis, emodin inhibited TWIST1-induced EMT via β-catenin/AKT pathway, and targeting these pathways could provide a novel therapeutic strategy for HNSCC patients [40]. In this study, we found that afatinib combined IR treatment improves epithelial-related proteins such as E-cadherin and claudin-1, while attenuating mesenchymal markers, including N-cadherin and snail. Furthermore, re-activation of Stat3 by IL-6 could promote the EMT process as well as cell migration and viability. Thus, this finding adds more evidence that the EMT process leads to radioresistance in NPC cells and demonstrates targeting EMT-associated regulators or signaling pathways might be applied as radiosensitizers for cancer patients to improve their prognosis effectively.

## 4. Materials and Methods

### 4.1. Cell Culture and CCK-8 Assay

5-8F and HNE2 cells were maintained in RPMI 1640 medium supplemented with 10% fetal bovine serum (FBS), penicillin (100 U/mL) and streptomycin (100 μg/mL). Cell Counting Kit 8 was used to conduct cell proliferation assay (CCK-8; Dojindo, Kumamoto, Japan). 5-8F and HNE2 cells (5 × 10^3^) were seeded in 96-well plates and induced with afatinib, IR or IL-6. Then, CCK-8 solution was added and incubated. Finally, the absorbance at 450 nm was tested by spectrophotometer.

### 4.2. Cell Migration and Wound Healing Assay

5-8F and HNE2 cells (2 × 10^5^) were pre-treated as the indicated group and suspended in serum-free medium. Cell suspensions (200 μL) was added in the upper culture chambers, while completed RPMI-1640 medium (600 μL) was added in the bottom chambers. After incubation for 48 h, the medium was ejected, and cells on the internal surface were wiped. Following, cells were fixed, stained, rinsed, and counted. To perform wound healing assay, NPC cells were seeded in 6-well plates and cell monolayer was scraped with a 10 μL pipette tip while cells were confluent. After incubation for 24 h, cell migration was observed via microscopy.

### 4.3. Immunoblotting

Cells were directly collected, and whole cell lysates (WCLs) were lysed in NP40 buffer supplemented with 20 mM β-glycerophosphate and 1 mM sodium orthovanadate. The following primary antibodies and reagents were used: Stat3 (60199-1-Ig, Proteintech, Rosemont, IL, USA), p-Stat3 (Tyr705) (YP0251, Immunoway, Plano, TX, USA), CD44 (60224-1-Ig, Proteintech, Rosemont, IL, USA), p-CD44 (Ser706) (YP0349, Immunoway, Plano, TX, USA), E-cadherin (YT1454, Immunoway, Plano, TX, USA), N-cadherin (YT2988, Immunoway, Plano, TX, USA), Snail (YT4351, Immunoway, TX, Plano, TX, USA), Claudin-1 (YT0942, Immunoway, Plano, TX, USA), ACTB (A5441, Sigma-Aldrich, St. Louis, MO, USA), Afatinib (S1011, Selleckchem.com, Houston, TX, USA), IL-6 (90107ES08, Yeasen, Shanghai, China).

### 4.4. Xenograft Mouse Experiment

The animal procedures were approved by the Ethical Committee for Animal Research. A total of 1 × 10^7^ cells (5-8F) in 100 μL PBS were injected subcutaneously into the right flank of each mouse. The formula, volume = [length × (width^2^)]/2, was used to analyze tumor volume. When tumor volume reached about 200 mm^3^, the mice were subjected to indicated treatments. Tumor sizes were monitored for about 14 days. Then, the mice were put to execution, and the tumors were obtained for HE and IHC staining.

### 4.5. Protein Extraction and Digestion

Cells were lysed in SDT buffer (4%SDS, 100 mM Tris-HCl, 1 mM DTT, pH7.6). The BCA Protein Assay Kit (Bio-Rad, Hercules, CA, USA) was used to quantify the protein amount. Protein digestion by trypsin was performed according to filter-aided sample preparation (FASP) procedure described by Matthias Mann. The digested peptides of each sample were desalted on C18 Cartridges (Empore™, St. Louis, MO, USA) SPE Cartridges C18 (standard density), bed I.D. 7 mm, volume 3 mL, Sigma, St. Louis, MO, USA), concentrated by vacuum centrifugation and reconstituted in 40 μL of 0.1% (*v*/*v*) formic acid.

### 4.6. Tandem Mass Tagging Labeling

The peptide mixture of each sample (100 μg) was labeled using TMT (Thermo Fisher Scientific, Waltham, MA, USA) reagent according to the manufacturer’s instructions. In short, one unit of TMT reagent was thawed and reconstituted in 24 μL acetonitrile (ACN). The peptide mixture was incubated in the TMT reagent. After 1 h, 200 μL of 5% hydroxylamine was used to quench. Then, the labeled peptide mixtures were pooled equally, desalted, and dried.

### 4.7. Phosphopeptide Enrichment and LC-MS/MS

The samples were reconstituted in 1.4 mL of precooled IAP Buffer. TiO_2_ beads were added into the mixture, shaked for 40 min, centrifuged and discarded the supernatant. The beads were put into the tips with stopper, then washed first with washing buffer 1 for 3 times, and then with washing buffer 2 for 3 times. The phosphopeptides were eluted with Elution buffer and concentrated under vacuum, then dissolved in 20 μL 0.1% formic acid for MS analysis.

LC-MS/MS analysis was conducted on a Q Exactive HF mass spectrometer (Thermo Scientific, Waltham, MA, USA) that was coupled to Easy nLC for 120 min. The peptides were loaded onto a reverse phase trap column (Thermo Scientific Acclaim PepMap100) connected to the C18-reversed phase analytical column (Thermo Scientific Easy Column) in buffer A (0.1% Formic acid) and separated with a linear gradient of buffer B (84% acetonitrile and 0.1% Formic acid) at a flow rate of 300 nL/min controlled by IntelliFlow technology. The mass spectrometer was operated in positive ion mode. MS data was acquired using a data-dependent top10 method dynamically choosing the most abundant precursor ions from the survey scan (300–1800 *m*/*z*) for HCD fragmentation. Automatic gain control (AGC) target was set to 3 × 10^6^ and maximum inject time to 10 ms. Dynamic exclusion duration was 40.0 s. Survey scans were acquired at a resolution of 70,000 at *m*/*z* 200 and resolution for HCD spectra was set to 17,500 at *m*/*z* 200, and isolation width was 2 *m*/*z*. Normalized collision energy was 30 eV and the underfill ratio, which specifies the minimum percentage of the target value likely to be reached at maximum fill time, was defined as 0.1%. The instrument was run with peptide recognition mode enabled.

### 4.8. Bioinformatic Analysis

Enrichment analyses were applied based on the Fisher’s exact test, considering the whole quantified proteins as background dataset. Benjamini–Hochberg correction for multiple testing was further applied to adjust derived *p*-values. Additionally, only functional categories and pathways with *p*-values under a threshold of 0.05 were considered as significant.

The protein sequences of the selected differentially expressed proteins were locally searched using the NCBI BLAST+ client software (ncbi-blast-2.2.28+-win32.exe) and InterProScan to find homologue sequences, then gene ontology (GO) terms were mapped and sequences were annotated using the software program Blast2GO. The GO annotation results were plotted by R scripts. Following annotation steps, the studied proteins were searched against the online Kyoto Encyclopedia of Genes and Genomes (KEGG) database (http://geneontology.org/, accessed on 10 December 2020) to retrieve their KEGG orthology identifications and were subsequently mapped to pathways in KEGG. The protein–protein interaction (PPI) information of the studied proteins was retrieved from IntAct molecular interaction database (http://www.ebi.ac.uk/intact, accessed on 11 March 2021) by their gene symbols or STRING software (http://string-db.org/, accessed on 11 March 2021).

### 4.9. Statistical Analysis

Statistical analysis was performed by unpaired, two-tailed Student’s *t*-test. A *p*-value < 0.05 is considered statistically significant. *, *p* < 0.05; **, *p* < 0.01; ***, *p* < 0.001.

## 5. Conclusions

Collectively, in this study, we discover that afatinib sensitizes NPC cells to radiation treatment and the underlying mechanisms. Taking the advantages of phosphoproteomics and bioinformatics analysis, we reveal that the phosphorylation statuses of some pivotal signaling pathways are robustly changed toward afatinib plus IR treatment. Furthermore, we demonstrate that afatinib enhances radiosensitivity by impeding the CD44-Stat3 axis and EMT process in vitro and in vivo. Herein, this work identifies a radiosensitive role of afatinib in NPC cells and establishes the phosphoproteomics profile and molecular details behind that. Whether afatinib regulates CSCs property when enhancing radiosensitivity in NPC remains unknown. Exploring such a question could be a promising field for future investigation. As for the clinical application, further studies should focus on the combination strategies of afatinib to improve the outcome of NPC patients.

## Figures and Tables

**Table 1:** Top 20 differentially expressed phosphoproteins (DEPs).

Upregulated DEPs (Top 20)				Downregulated DEPs (Top 20)			
ID	Proteins	FC Value	*p* Value	ID	Proteins	FC Value	*p* Value
P20020	ATP2B1	1.764	0.048	Q04637	EIF4G1	0.4	0.006
Q13459	MYO9B	1.517	0.009	Q04637	EIF4G1	0.429	0.018
Q9H8Y8	GORASP2	1.49	0.014	Q15648	MED1	0.467	0.014
Q5VT25	CDC42BPA	1.481	0.033	P16070	CD44	0.475	0.011
Q9Y570	PPME1	1.466	0.027	P40763	STAT3	0.476	0.029
O94921	CDK14	1.46	0.004	Q86X27	RALGPS2	0.497	0.012
Q14669	TRIP12	1.443	0.02	Q7RTP6	MICAL3	0.504	0.008
O15355	PPM1G	1.432	0.007	Q09666	AHNAK	0.509	0.043
Q9H3H3	C11orf68	1.429	<0.001	Q9H2G2	SLK	0.512	0.007
P18433	PTPRA	1.423	0.006	Q9BRS8	LARP6	0.524	0.021
P13796	LCP1	1.401	0.047	Q9H0X4	FAM234A	0.526	0.02
Q12802	AKAP13	1.386	0.005	Q01196	RUNX1	0.535	0.025
Q9NP64	ZCCHC17	1.37	0.007	P80723	BASP1	0.543	0.033
P53999	SUB1	1.365	0.021	Q09666	AHNAK	0.545	0.01
Q9H2H9	SLC38A1	1.364	0.004	Q13555	CAMK2G	0.546	0.049
Q8WX93	PALLD	1.363	0.01	Q5T5U3	ARHGAP21	0.558	0.003
Q14160	SCRIB	1.362	0.016	Q9Y520	PRRC2C	0.565	0.001
Q9UQ35	SRRM2	1.355	0.016	Q5T5U3	ARHGAP21	0.594	0.022
Q96L91	EP400	1.354	0.015	Q9NUQ3	TXLNG	0.596	0.001
Q96MU7	YTHDC1	1.349	0.034	Q04637	EIF4G1	0.602	0.002

## Data Availability

The mass spectrometry proteomics data have been deposited to the ProteomeXchange Consortium (http://proteomecentral.proteomexchange.org) (accessed on 19 December 2022) with the dataset identifier PXD037010.

## Abbreviations

EMT	Epithelial-Mesenchymal Transition
NPC	Nasopharyngeal carcinoma
EBV	Epstein–Barr virus
CSCs	Cancer stem cells
NSCLC	Non-small cell lung cancer
PTMs	Protein posttranslational modifications
DEPs	Differentially expressed proteins
